# Supplementary material for: Effects of Isoflavone-Rich NADES Extract of Pueraria lobata Roots and Astaxanthin-Rich Phaffia rhodozyma Extract on Prostate Carcinogenesis in Rats
Source: Plants (Basel). 2023 Jan 26;12(3):564. doi: 10.3390/plants12030564 (PMC9920470; doi:10.3390/plants12030564)
Supplement: Supplementary file 1 [file plants-12-00564-s001.zip › plants-2088581-supplementary.pdf]

Table S1. Blood count parameters

Group	WBC 10^9/L	Lymph# 10^9/L	Mon# 10^9/L	Gran# 10^9/L	RBC 10^12/L	HGB g/L	HCT %	PLT 10^9/L
IC	12.2 ± 0.9	7 ± 0.5	0.44 ± 0.04	3.5 ± 0.3	9.1 ± 0.1	163 ± 2	47.1 ± 0.5	791 ± 42
PCa	13.4 ± 0.5	7 ± 0.4	0.53 ± 0.02	5.8 ± 0.4 [a]	9.4 ± 0.1	167 ± 1	48.6 ± 0.3	615 ± 42 [a]
PCa + isoflavones	13.1 ± 0.6	6.7 ± 0.3	0.46 ± 0.03	5.6 ± 0.5 [a]	9.3 ± 0.1	169 ± 2	48.9 ± 0.8	810 ± 48 [b]
PCa + astaxanthin	13.1 ± 0.7	6.4 ± 0.2	0.58 ± 0.04	5.1 ± 0.4 [a]	9 ± 0.2	160 ± 4	47.1 ± 1	783 ± 69 [b]

[a] – $p < 0.05$, compared to IC group; [b] – $p < 0.05$, compared to PCa group

Table S2. Blood biochemistry parameters

Group	Total Protein, g/l	Albumin, g/l	Globulin, g/l	Albumin/ Globulin ratio	LDH, U/l	Amylase, U/l	Alkaline Phosphatase, U/l	AST, U/l	ALT, U/l	Ast/Alt ratio
IC	66.6 ± 1.5	29.5 ± 0.9	37.1 ± 1.1	0.80 ± 0.04	1703 ± 197	1693 ± 161	133 ± 16	150 ± 13	80.6 ± 4.8	1.86 ± 0.13
PCa	62.2 ± 1.8	29.1 ± 1.1	33.1 ± 1.5	0.91 ± 0.06	2045 ± 161	1675 ± 97	152 ± 24	164 ± 17	87 ± 6.2	1.89 ± 0.12
PCa + isoflavones	65.9 ± 2.5	32.0 ± 1.0	33.9 ± 2.2	0.99 ± 0.07	2113 ± 305	1489 ± 114	135 ± 26	137 ± 4	76.1 ± 3.7	1.82 ± 0.06
PCa + astaxanthin	66.8 ± 2.5	31.7 ± 1.3	35.1 ± 1.5	0.91 ± 0.04	1562 ± 131	1702 ± 86	188 ± 22	141 ± 10	77.8 ± 7.4	1.85 ± 0.15

Table S2, Continuation

Group	Glucose, mmol/l	Triglycerides, mmol/l	Total bilirubin, µmol/l	Direct bilirubin, µmol/l	Creatinine, µmol/l	Urea, mmol/l	Magnesium, mmol/l	Iron, µmol/l
IC	4.36 ± 0.22	1.43 ± 0.36	1.72 ± 0.31	0.25 ± 0.04	53.1 ± 2.2	4.95 ± 0.3	0.73 ± 0.02	23.1 ± 1.6
PCa	4.5 ± 0.21	1.38 ± 0.29	1.75 ± 0.31	0.27 ± 0.08	47.2 ± 4.1	5.1 ± 0.32	0.78 ± 0.01	26.6 ± 1.8
PCa + isoflavones	4.75 ± 0.17	1.14 ± 0.12	2.22 ± 0.36	0.38 ± 0.07	47 ± 1.6	5.24 ± 0.19	0.78 ± 0.03	29.4 ± 2.3
PCa + astaxanthin	5.05 ± 0.28	1.55 ± 0.34	2.2 ± 0.3	0.19 ± 0.1	51.7 ± 2.7	5.46 ± 0.24	0.75 ± 0.03	28.1 ± 2.7

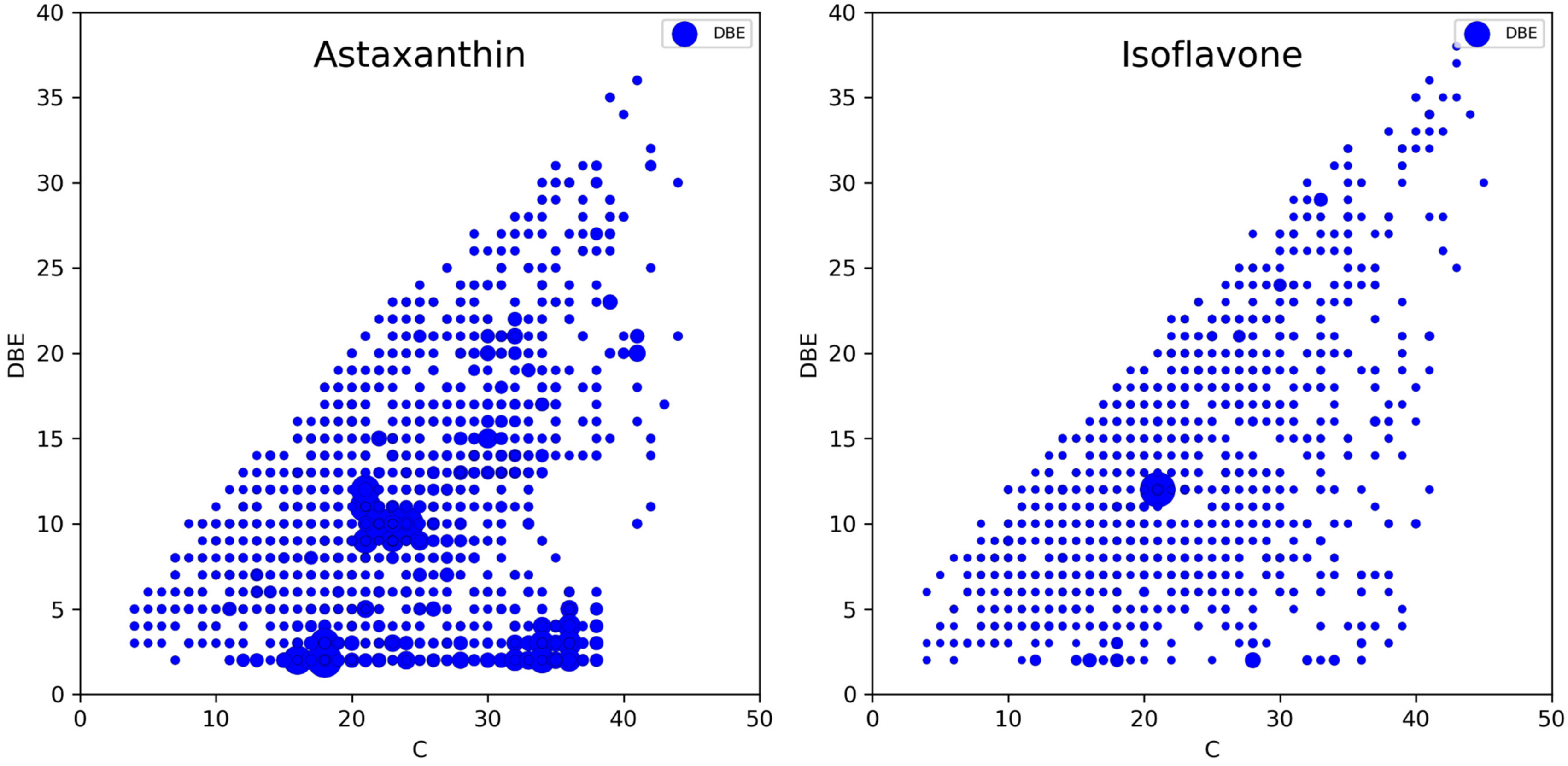

Figure S1. DBE vs C scatter plot representing the distribution of molecular components with different unsaturation. Size of dots correspond to relative intensity

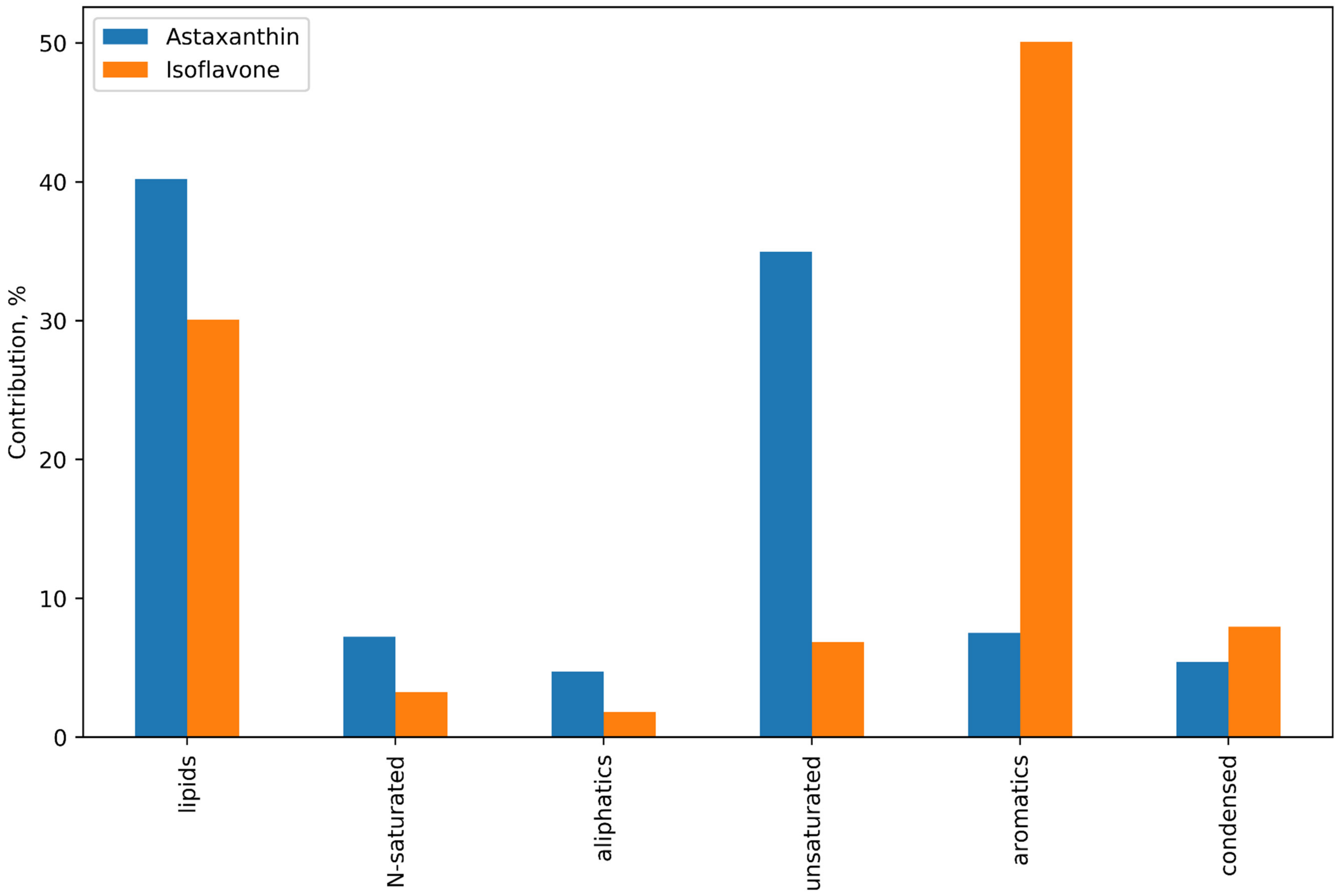

Figure S2. Contribution of AI_{con}-based chemical classes to molecular composition of samples
